# A new turbidimetric immunoassay for serum calprotectin for fully automatized clinical analysers

**DOI:** 10.1186/s12950-015-0090-3

**Published:** 2015-07-25

**Authors:** Tom Nilsen, Kathrin Sunde, Anders Larsson

**Affiliations:** Gentian Technology AS, Moss, Norway; Department of Medical Sciences, Uppsala University Hospital, Uppsala, Sweden

## Abstract

Serum and plasma calprotectin concentration is shown to be elevated when neutrophils are activated, and may therefore be used as a marker for inflammatory diseases. A serum calprotectin immunoassay was developed based on calprotectin values observed in samples from the intensive care unit. The polyclonal avian antibodies were raised and affinity purified with calprotectin antigens. The performance was tested and it was observed that the assay was linear in the range 0.3–24.7 mg/L, the limit of quantitation was observed to be lower than 0.3 mg/L, no antigen excess was observed up to 54 mg/L, all CVs were lower than 1.8 % in the precision study, the calibration curve stability was longer than 6 weeks, and there was no significant interference detected for haemoglobin, intralipid or bilirubin. The serum calprotectin immunoassay presented in this paper performs well within the criteria carefully set from the limited clinical experience obtained in both serum and plasma. In addition it is commutable with Bühlmann MRP8/14 ELISA.

## Background

In clinical practice, the diagnosis of a bacterial infection requiring antibiotic treatment is made from observation of clinical symptoms, supported by leukocyte counts and/or C-reactive protein (CRP) measurement. It has been estimated that about 40 % of cases are misclassified between bacterial infections and other causes [1]. Previously it has been shown that the neutrophil activation marker HNL/NGAL could distinguish between acute bacterial and viral infections with a sensitivity and specificity of more than 95 % [1].

Similar to HNL/NGAL, calprotectin is a neutrophil activation marker. The plasma concentration of calprotectin is higher than for HNL/NGAL. It is therefore easier to develop calprotectin assays that can be applied on standard chemistry analysers. Calprotectin is mostly found in the neutrophils where it accounts for approximately 30–40 % of the cytosol protein content. Calprotectin has been used as a marker for neutrophil activation and has been shown to be elevated in inflammatory conditions where neutrophil activation is involved [2]. Elevated levels of calprotectin have been detected in blood and faeces, and calprotectin is considered to be an important inflammation marker [3, 4]. The calprotectin molecule comprises of two members of the S100 family; S100A8 (MRP8) and S100A9 (MRP14). Calprotectin thus contains these two subunits, but the exact *in vivo* structure of calprotectin is debated. Johne *et al.* suggest it is a heterotrimer [5], Pepper *et al.* suggest it is a heterodimer [6], and Steinbakk *et al.* suggest it is a hetero-tetramer when calcium is present [7]. In faeces, elevated calprotectin concentrations are used as markers for intestinal inflammatory diseases, such as inflammable bowel disease (IBD) and Crohn’s disease among others [8]. Fecal-calprotectin is widely used to discriminate between IBD and irritable bowel syndrome and the test is thus used in large volumes. Serum calprotectin is not as widely used as faecal calprotectin, but it has also been used as an inflammatory marker. It has been reported to be a valuable marker for sepsis [6, 9], and a marker for acute appendicitis [10]. Bacterial infections usually cause neutrophil activation, thus, in theory a neutrophil activation marker should be more specific for bacterial infections than e.g. CRP. The rapidly growing problem with antibiotic resistance has resulted in demands for more specific use of antibiotics. It is thus important to find markers that can better distinguish between bacterial and viral infections. Sepsis patients may have low levels of neutrophils in the blood as the neutrophil activation causes the neutrophils to attach to the endothelium and leave the circulation. This can be circumvented by using a protein marker for neutrophil activation rather than measuring the number of circulating neutrophils. ELISA is the most used method for measuring Calprotectin, but is usually associated with long test turnaround times. In this study we have used the Calprotectin ELISA kit from Bühlmann Laboratories AG, Schönenbuch, Switzerland for comparison of patient results. ELISAs are usually also more expensive than turbidimetric assays as they are more labour intensive. Laboratories collect samples to fill up entire microtiter plates, which could take several days, in order to reduce reagent and labour costs. A particle enhanced turbidimetric immunoassay are usually used as a random access test and the samples will be run continuously as they arrive at the laboratory which will contribute to reduced test turnaround times. The immunoparticles developed for the Gentian Serum Calprotectin immunoparticles were coated with polyclonal avian antibodies. The antibodies were raised in hens which were immunised with purified MRP8/MRP14 heterodimers. The polyclonal antibodies are assumed to detect both monomers and dimers and even oligomers of the MRP8/MRP14 complex. Avian antibodies do not react with rheumatoid factors, human anti-mouse IgG antibodies (HAMA) or the human complement system. These are all well-known causes of erroneous test results in tests based on mammalian antibodies. The aim of the present study was to develop and validate a particle enhanced turbidimetric immunoassay (PETIA) for the detection of human calprotectin in serum or plasma.

## Materials and methods

### Reagents

The Gentian Serum Calprotectin Immunoassay reagents consist of immunopartcles, buffer, calibrators and controls.

### Immunoparticles and reaction buffer

The Gentian Serum Calprotectin immunoparticles were prepared by covalently attaching purified avian immunoglobulin fractions to uniform polystyrene particles. The avian immunoglobulin fractions were purified from eggs from immunized hens. The antibodies were directed against human calprotectin (hetero dimer of MRP8/MRP14). Calprotectin extracted and purified from human granulocytes was used in the process of raising the avian antibodies. The reaction buffer was a 3-(N-morpholino)propanesulfonic (MOPS) buffer with pH = 7.2.

### Calibrators

Purified calprotectin was diluted in a phosphate buffer pH = 7.4 to achieve six calibration levels: 0, 1, 3, 6, 15, 30 mg/L. The calprotectin concentration of the stem solution was assigned by the Biuret method (Bioquant™, Merck KGaA, Darmstadt, Germany).

### Controls

Controls were prepared by adding purified calprotectin to normal human serum. Two levels were prepared; 1.2 and 8.9 mg/L.

### Instruments

The fully automated clinical chemistry instrument Mindray™ BS-380 (Mindray Medical International, Shenzhen, China) was used for all calprotectin measurements.

Parameter settings were optimised for the Gentian Serum Calprotectin Immunoassay on this instrument. For the method comparison the Gentian Calprotectin Immunoassay was compared to a MRP8/MRP14™ ELISA kit from Bühlmann Laboratories AG, Schönenbuch, Switzerland.

### Applications/Assay procedures

A set of parameter settings were optimised for the Gentian Serum Calprotectin Immunoassay on Mindray BS380(™), Shenzhen, China. The parameter settings were: sample volume = 3 μL, R1 volume = 200 μL and the R2 volume = 30 μL. The wavelength was 605 nm and the reading times 38/39-55/56, which is equal to 204 s as each cycle counts 12 s.

### The studies

To demonstrate the performance characteristics of the Gentian Serum Immunoassay, a calibration curve was established using the settings described above and controlled by measuring the QC’s the same day as the studies were performed, unless otherwise specified. The studies were based on the CLSI protocols.

### Limit of quantitation (LoQ)

Four samples at different concentration levels ranging from 0.2 to 0.6 mg/L were prepared by diluting a normal serum sample in the calibrator matrix, these were aliquoted into 12 tubes of 300 μL and stored at −50 °C. The samples were brought to room temperature, vortexed and measured directly. Four tubes of each concentration level were measured in 4 replicates for 3 days. In total, 36 measurements for each concentration level were performed over 3 days with a recalibration and QC measurements every day. The undiluted sample was measured in 12 replicates the first day in order to calculate the theoretical concentration values of the diluted samples.

CV’s and biases from theoretical values were calculated, and the total error results were decided by the root mean square method. LoQ was determined to be the lowest calprotectin concentration sample where the total error was lower than 20 %.

### Antigen excess

Samples were prepared by spiking a pool of normal serum with granulocyte extract to a calprotectin concentration of approx 50 mg/L. A dilution series of 9 samples was made from this spiked pool, ranging from 2.5 to 100 % of the original sample. All samples were measured in duplicate. The antigen excess occurs at the lowest concentration where the returned turbidimetric results are below the value of the turbidimetry result of the highest calibrator. A result above the highest calibration point will force the instrument to flag the sample and the user will be notified by alarm to start a diluted rerun if needed.

### Linearity

The preparation of the linearity series was carried out by preparing a high calprotectin serum sample and a low calprotectin serum sample. The high sample was prepared by spiking a normal serum sample, to a concentration of 20–30 mg/L, with granulocyte extract containing high levels of calprotectin. The low sample was prepared by diluting a normal serum sample with saline to a concentration below 0.5 mg/L. A dilution series was prepared by mixing the high serum sample with the low serum sample in the range from 100 to 0 %. Ten samples in the series were prepared. The 100 % serum sample and the 0 % serum sample were measured in triplicate in order to get assigned values. The expected value of each sample in the series was then calculated from the assigned values and dilution factors. All samples were measured in duplicates. The deviation from expected values was calculated. The acceptable deviation from the theoretical values was set to be less than 10 %.

### Precision and calibration curve stability

Three samples, and 2 controls (low and high), were dispensed into 14 aliquots of 300 μL, and stored in cryo tubes below −55 °C, the concentrations of these spanned the calibration curve. At day one the calibration curve was established. The samples were brought to room temperature, vortexed and measured immediately in duplicate. This procedure was repeated for 10 subsequent working days, without recalibration. Total CV’s were calculated. The reagents were stored on-board during the study period. The established calibration curve was further applied to measure the same samples every week for up to 4 more weeks after the last day of the precision study to investigate the calibration curve stability of the immunoassay.

### Interference

The purpose of this study was to determine if haemoglobin, intralipid or bilirubin interfere with the calprotectin immunoassay on Mindray BS-380™.

The goal was to demonstrate that the assay does not interfere with these substances at the following concentrations: Bilirubin up to 400 mg/L, Haemoglobin up to 5 g/L, Intralipid up to 10 g/L. The haemoglobin samples were prepared by adding 0.106 ml of 94 g/L hemolysate to 1.894 ml of serum. The control sample was prepared by adding 0.106 ml of saline in 1.894 ml of serum. No significant interference is defined as less than 10 % difference between test and control samples.

### Method comparison

In total 60 samples were measured on both Gentian Calprotectin Immunoassay and Bühlmann ELISA the same day. Bühlhmanns ELISA has a measuring range from 0.4 to 24 mg/L. The samples measured were distributed over the ELISA calibration curve with a predominance of samples between the lower reference interval up to approximately 6.5 mg/L. The samples were first measured on the Gentian Serum Calprotectin Immunoassay and then immediately measured on the ELISA. The results were evaluated using Analyse-It(™) for Excel, Analyse-It Software, Ltd, Leeds UK. For the ELISA the duplicates could vary significantly. The observed replicates that deviated by more than 25 % were defined as outliers and removed from the data set.

### Lot variation

Two immunoparticle batches were prepared with two different antibody batches, where the antibodies were raised using two separately prepared antigens. The results were evaluated using Analyse-It™ for Excel, (Analyse-It Software, Ltd, Leeds UK).

### Patient samples

P-calprotectin was analyzed in Li-heparin plasma from 50 blood donors; 17 females (age 18–65) and 33 males (age 21–75 years) and 13 patients with sepsis; three females (age 26–45) and ten males (age 40–88 years).

## Results

### Limit of quantitation

The lower quantitation limit (LoQ) was defined as the lowest sample with a total error less than 20 %. For the serum calprotectin assay the LoQ was observed to be 0.35 mg/L. The total error for this sample was 15.9 %. Table 1 shows the results from all samples tested.

**Table 1:** Determination of the lower limit of quantitation of the calprotectin assay

Mean (mg/L)	1,316	0,624	0,489	0,347	0,206
std (mg/L)	0,0198	0,0340	0,0336	0,0400	0,0344
CV (%)	1,5	5,4	6,9	11,5	16,7
Theoretical value (mg/L)	1,32	0,66	0,53	0,40	0,26
Deviation from theoretical value (mg/L)	−0,004	−0,036	−0,039	−0,049	−0,058
TE	0,0202	0,0494	0,0518	0,0631	0,0678
TE%	1,5	7,5	9,8	15,9	25,7

The results are presented as CV and deviation from theoretical value. The lowest concentration with an acceptable total error was 0.37 mg/L

### Antigen excess

**Fig. 1:**
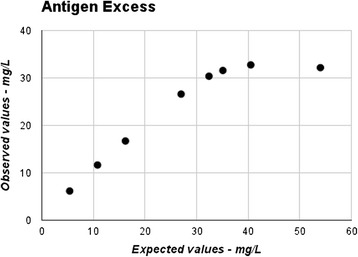
Measured values of a diluted series made from a high concentration sample spiked to 54 mg/L. All samples with concentrations between 30 mg/L and 54 mg/L is measured to be above the highest calibration standard and will therefore go to rerun automatically. Hence there is no antigen excess observed for samples up to 54 mg/L

The prepared series were measured in duplicate and the mean values were plotted in Fig. 1. All observed results from sample concentrations in the range 30−54 mg/L were above the highest calibrator value, which was 30 mg/L. No antigen excess from concentrations below 54 mg/L was observed.

### Linearity

**Fig. 2:**
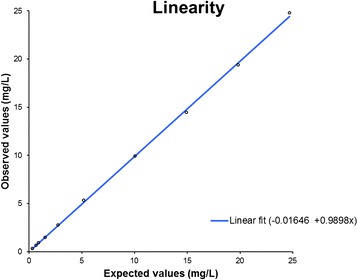
The observed data from the prepared linearity series with a linear fit. The observed values measured on a dilution series prepared by diluting a high calprotectin concentration serum sample with a low concentration serum sample were compared to the expected values. The observed values are on the y-axis and the expected on the x-axis. The expected values were calculated from dilution factors and the assigned values to the high sample (24.7 mg/L) and the low sample (0.3 mg/L)

There were no observations deviating more than 5 % from expected values when measuring the prepared linearity series. The observed data are presented in Fig. 2 and Table 2. This is within the predefined criteria of ±10 % from the expected values calculated from the assigned values of the 0 % sample and the 100 % sample, and the dilution factors.

**Table 2:** Linearity study by diluting a sample with a high calprotectin concentration in a sample with low calprotectin concentration

% high sample	Expected values (mg/L)	Observed values (mg/L)	Recovery from expected values (%)
100	24,70	24,76	100,2
80	19,82	19,40	97,9
60	14,95	14,46	96,7
40	10,07	9,90	98,3
20	5,20	5,34	102,8
10	2,76	2,77	100,3
5	1,54	1,48	95,9
2,5	0,93	0,91	97,9
1,5	0,69	0,66	95,8
0	0,32	0,31	96,9

The observed values were compared with the expected values and the recovery was calculated. The observed data from the linearity series deviated less than ±5 % from the expected values calculate from the assigned values of the 0 % sample and the 100 % sample and the dilution factors

### Precision

The 10 day precision study showed CVs lower than 1.5 % for all samples ranging from 1.17 mg/L up to 22.14 mg/L. The samples were measured on 10 subsequent working days. The data are presented in Table 3.

**Table 3:** The daily means, total means, standard deviations and total CV’s from the precision study

Day no:	Daily mean P1	Daily mean P2	Daily mean P3	Daily mean P4	Daily mean P5
Day 1	1,17	6,82	22,65	1,22	8,78
Day 2	1,16	6,64	22,25	1,21	8,57
Day 3	1,17	6,68	22,11	1,19	8,55
Day 4	1,18	6,69	22,27	1,22	8,56
Day 5	1,19	6,67	22,14	1,19	8,55
Day 6	1,17	6,66	22,27	1,17	8,59
Day 7	1,17	6,68	21,98	1,20	8,62
Day 8	1,16	6,66	21,83	1,19	8,60
Day 9	1,21	6,73	21,95	1,22	8,59
Day 10	1,18	6,69	22,03	1,18	8,61
Total mean	1,17	6,69	22,14	1,20	8,60
Total Stdev	0,0147	0,0523	0,2303	0,0174	0,0680
Total CV	1,3	0,8	1,0	1,4	0,8

### Calibration curve stability

**Fig. 3:**
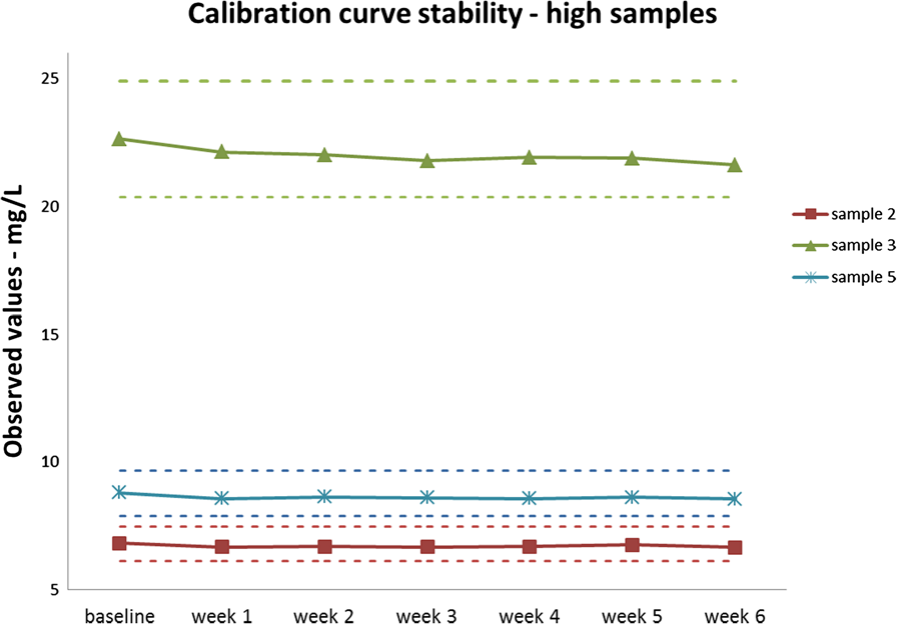
Samples 2, 3 and 5 measured within the range of baseline ±10 % for all 6 weeks. From the observed results, the tubidimetric serum calprotectin immunoassay can claim 6 week of calibration curve stability for samples higher than 6 mg/L

**Fig. 4:**
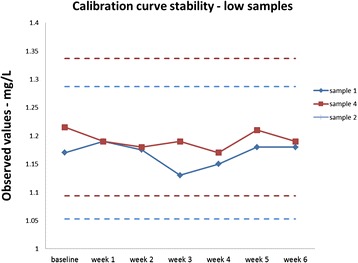
Samples 1 and 4 measured within the range of baseline ±10 % for all 6 weeks. From the observed results, the turbidimetric serum calprotectin immunoassay can claim 6 week of calibration curve stability for samples higher than 6 mg/L

The observation of the measured samples over time, shown in Figs. 3 and 4, suggests a calibration curve stability of at least 6 weeks. The total CV for all samples was less than 2 %. The concentration of samples 1, 2, 3, 4 and 5 were 1.17, 6.82, 22.65, 1.22 and 8.78 mg/L respectively at baseline.

### Method comparison

**Fig. 5:**
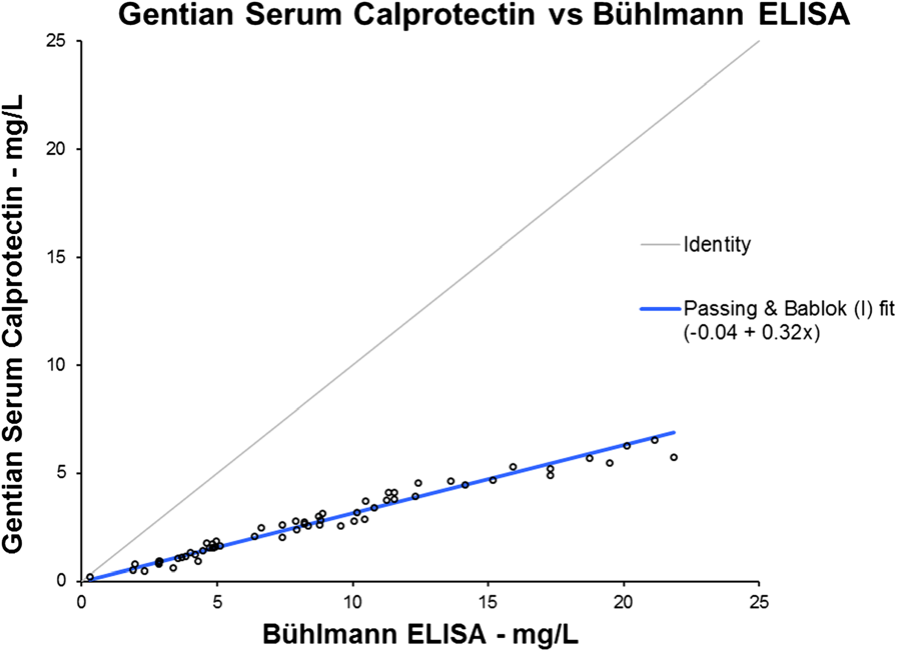
60 samples were measured on both Bühlmann MRP8/MRP14 ELISA and the turbidimetric serum calprotectin immunoassay. The comparison with the results from the ELISA on the x-axis and the results from the turbidimetric assay on the y-axis shows that the two methods correlate well and are highly commutable

**Fig. 6:**
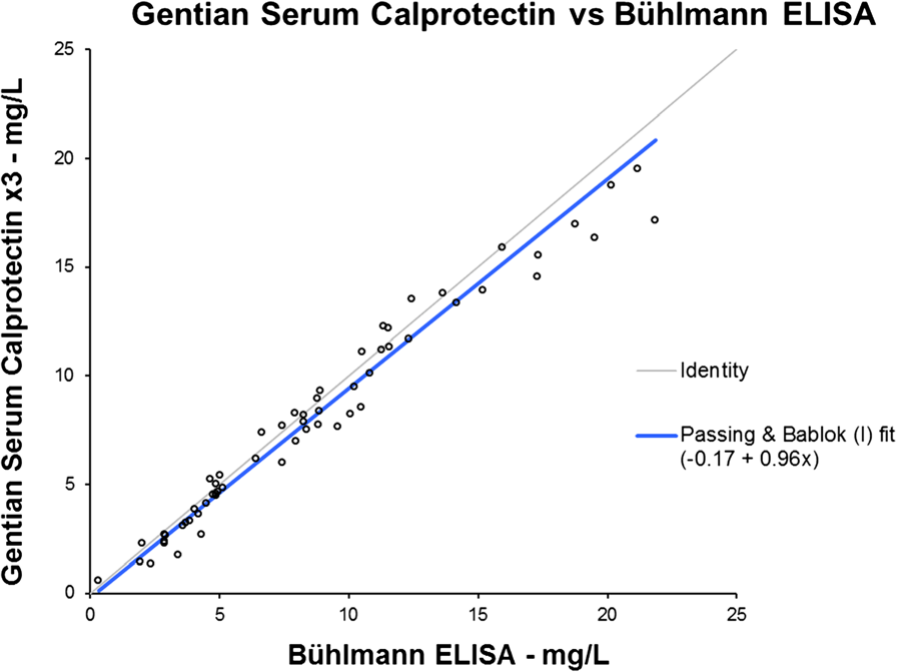
A factor 3 was multiplied by the results from the turbidimetric assay. The results were then compared to the results from the Bühlmann MRP8/MRP14 ELISA. Applying a factor 3 to the data measured on Gentian’s method improved the Passing Bablok linear regression fit and demonstrates the commutability between the methods

The method comparison data shows that the tubidimetric calprotectin immunoassay is commutable with the Bühlmann ELISA, see Fig. 5. In order to demonstrate the commutability with a better Passing Bablok regression fit, it was appropriate to apply a factor of 3 on the Gentian data. As shown in Fig. 6, the regression fit was then acceptable with an intercept of −0.17 and a slope of 0.96.

### Lot variation

**Fig. 7:**
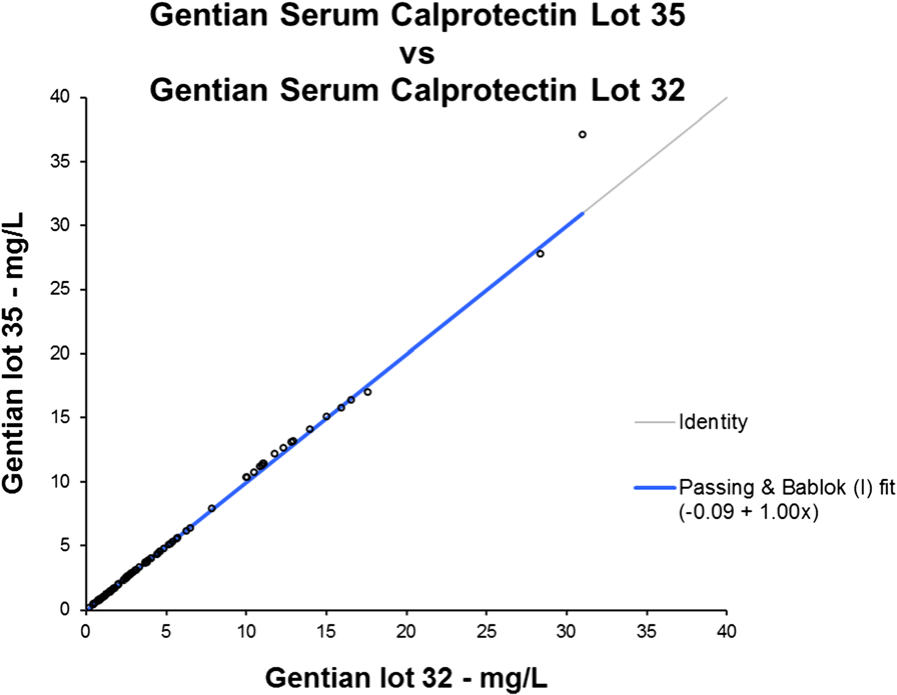
Comparing two preparations with two different batches of antibodies raised and purified with different calprotectin antigens in different hen batches, indicates a low lot to lot variation. The results from the two batches of immunoparticles correlate well

The two batches of immunoparticles prepared with two different antibodies, raised against different antigen preparations, correlate well. Figure 7 shows the data with Passing Bablok regression fit. The equation of the fit was 1.00× − 0.09.

### Interference

None of the three substances interfere significantly at tested levels, see Table 4. The maximum allowed difference between the test and control samples was decided to be 10 % from the value of the control sample.

**Table 4:** Interference of haemoglobin, intralipid and bilirubin was tested by adding these substances to patient samples

Substance	Amount of substance	Control - mg/L	Test - mg/L	Observed difference - mg/L	Max allowed difference - mg/L	Within acceptance (yes/no)
Hemoglobin - low	5 g/L	1,54	1,46	0,08	0,15	yes
Hemoglobin - high	5 g/L	10,06	9,92	−0,14	0,99	yes
Intralipid - low	10 g/L	1,48	1,37	−0,11	0,15	yes
Intralipid - high	10 g/L	9,71	9,7	−0,01	0,97	yes
Bilirubin - low	400 mg/L	1,52	1,5	−0,02	0,15	yes
Bilirubin - high	400 mg/L	10,02	10,01	−0,01	1,00	yes

The calprotectin values before and after addition was compared. A difference of ≤10 % was the criteria for acceptance. None of the substances had significant interference at tested levels

### Analysis of patient samples

The median P-calprotectin value in the blood donor group was 0.80 mg/L (Interquartile range (IQR) 0.37–1.19 mg/L) while the median P-calprotectin value in the sepsis group was 4.18 mg/L (IQR 2.51–8.32). The difference between groups was significant according to Mann–Whitney *U* Test (*p* = 0.003).

## Discussion

Antibiotic resistance is generally accepted as one of the greatest threats to our health. It is thus essential to slow the emergence of resistance and extend the useful lifetime of antibiotics. A goal set for 2020 by the United States Centers for Disease Control and Prevention (CDC) is a reduction of inappropriate antibiotic use by 50 % in outpatient settings and by 20 % in inpatient settings [11]. To be able to reach this goal we need new markers that accurately can distinguish between bacterial and viral infections. Identification of a biomarker able to clearly distinguish bacterial from non-infectious systemic inflammation is imperative. The development of new biomarkers takes several years and it is thus important that we now start the development and evaluation of new markers. Neutrophil activation markers such as calprotectin, alone or in combination with other inflammation markers, could potentially be superior to e.g. CRP to distinguish between bacterial and viral infections.

Sepsis is an acute condition that requires rapid treatment. If new markers are to be useful they need to be available with short test turn-around times at all hours. It is also important that the tests can be run at as many hospitals as possible either as centralized assays or as POC tests. The purpose of this paper is to validate the technical performance of a new serum calprotectin turbidimetric immunoassay, which hopefully will make measurement of serum calprotectin more available. The method can easily be applied on the chemistry analyzers that are standard instrumentation in hospital laboratories. As this is a new assay it was important to validate the performance of the turbidimetric assay. It is important that the measuring range is suitable for patient samples to avoid excessive reruns or antigen excess problems. The most severe cases of sepsis are usually treated in the intensive care unit. To verify the suitability of the measuring range we analyzed consecutive samples from the intensive care unit. None of the samples had calprotectin values higher than 25 mg/L and we did not observe any antigen excess problems. The assay measured accurately down to 0.35 mg/L as shown in the LoQ study. In an earlier published study [12], the reference interval of serum calprotectin was found to be 0.5–2.5 mg/L. Thus, the majority of patient samples will be within the measuring range. Samples above 30 mg/L up to 54 mg/L all measured above the highest calibrator standard, which was 30 mg/L. This means there is no risk of antigen excess problems up to 54 mg/L. During this project there were no patient samples observed above 29 mg/L, based on this experience, the assay will most likely not run into problems when it comes to antigen excess. There were no significant interferences by haemoglobin, lipids or bilirubin and the coefficient of variations were below 1.8 % which is lower than for most ELISA methods. Gentian serum calprotectin assay is based on polyclonal avian antibodies in contrast to Bühlmanns MRP8/MRP14 ELISA, which is based on mammalian monoclonal antibodies. This can explain some of the spread in the method comparison plot. Polyclonal antibodies will be able to detect and add to the signal any composition of MRP8/MRP14 complexes. Monomers will for instance to some degree be detected and add signal to the measurement. The monoclonal based ELISA will only detect and measure the dimer MRP8-MRP14 according to the manufacturer. There is no international standard for serum calprotectin, which forces the manufacturers to rely on internally established standards to avoid lot to lot variation. Since the method to assign value to these internal standards is of free choice, there will be different levels of the standards among the manufacturers depending on which method they choose. We believe this is the cause of the difference between the measured values of Gentian’s assay and the Bühlmann ELISA. The commutability shown in the method comparison study demonstrates that these two methods can easily be adjusted to measure on the same levels if required.

## Conclusions

The serum calprotectin immunoassay presented in this paper performs well within the criteria. With this assay it should not be expected to observe any antigen excess, sensitivity limitations or linearity problems within any part of the observed measuring range. The precision is good and the there is no significant interference observed with haemoglobin, bilirubin or intralipid. The method comparison shows that the method is commutable with Bühlmann MRP8/14 ELISA and will correlate well when the calibrators are adjusted to an international standard in the future.
